# Simultaneous Open Reduction and Corrective Osteotomy for a Child With Lateral Condylar Fracture and Cubitus Varus Deformity Following Supracondylar Fracture: A Case Report

**DOI:** 10.7759/cureus.26796

**Published:** 2022-07-12

**Authors:** Koichi Yano, Yasunori Kaneshiro, Takuya Yokoi, Hideki Sakanaka

**Affiliations:** 1 Department of Orthopedic Surgery, Seikeikai Hospital, Sakai, JPN

**Keywords:** secondary fracture, corrective osteotomy, lateral condylar fracture, supracondylar fracture, cubitus varus deformity

## Abstract

Posttraumatic cubitus varus deformity in pediatric patients may cause second fractures of the distal humerus. Corrective osteotomy is used to obtain good alignment and is generally performed for patients with prolonged deformity or bony union after fracture. We report the case of a 10-year-old boy who presented with elbow pain after falling. Plain radiography showed lateral condylar fracture and cubitus varus deformity. This injury was the fourth fracture of the same distal humerus. Open reduction and internal fixation for lateral condylar fracture and lateral closing wedge osteotomy for cubitus varus deformity were performed simultaneously. At the last follow-up, one and a half years after operation, plain radiography showed closure of the physis of the distal humerus, and coronal alignment was maintained. The patient was asymptomatic and satisfied with cosmetic issues. There was no fracture after two surgical procedures simultaneously.

## Introduction

Cubitus varus deformity is a complication of pediatric distal humeral fractures such as supracondylar fractures, lateral condyle fractures, and physeal fractures [1-5]. This deformity causes future complications such as tardy ulnar nerve palsy, posterolateral rotatory instability, and second fracture of the distal humerus [1,5-8].

Generally, corrective osteotomy for posttraumatic cubitus varus is performed for patients with prolonged deformity or bony union after fracture [1,5]. To our knowledge, there are only a few reports in the English literature of surgical treatments of both open reduction for fracture treatment and corrective osteotomy simultaneously to prevent a sequential fracture following cubitus varus deformity.

We report a pediatric case of a fourth fracture, lateral epicondylar fracture, due to a posttraumatic cubitus varus deformity which was treated by means of open reduction and internal fixation (ORIF) for fracture and lateral closing-wedge osteotomy simultaneously, the surgical technique, and the postoperative outcomes.

## Case presentation

**Figure 1:**
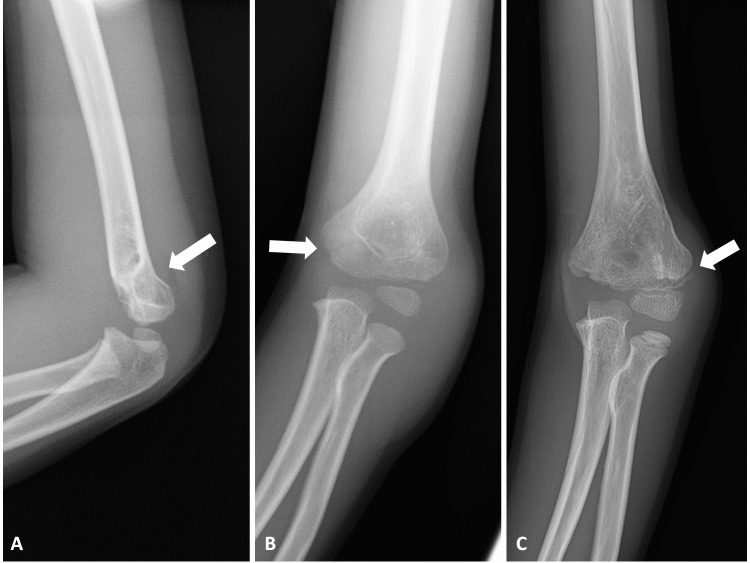
Plain radiography of the serial fractures at the left elbow. (A) The first fracture at the age of four years. (B) The second fracture at the age of five years. (C) The third fracture at the age of eight years. (Arrows: fracture sites).

A 10-year-old, right-handed boy fell and landed on his left hand while walking and complained of left elbow pain. He had previously fractured the same left distal humerus three times; the treatments were conservative for supracondylar fracture at the age of four years caused by falling from bicycle (Figure 1A), closed reduction, and percutaneous pinning for supracondylar fracture at the age of five years caused by falling down and stumbling on the step (Figure 1B), and again conservative for lateral condylar fracture at the age of eight years caused by fall while playing skateboard (Figure 1C).

**Figure 2:**
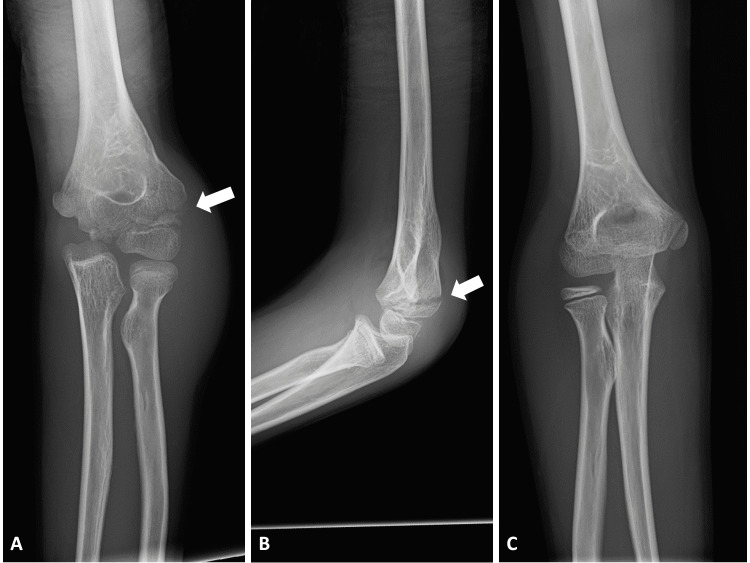
Plain radiography of the fourth fracture. (A) Anteroposterior and (B) lateral view of the left elbow. (C) Anteroposterior view of the uninjured right elbow. (Arrows: fracture site).

Initial examination showed swelling and tenderness on the lateral side of the left elbow without a neurovascular disorder. Plain radiographs of the left elbow showed lateral condylar fracture, 7 mm displacement of the fragment in pronation-oblique view, and cubitus varus deformity (Figures 2A-2C).

Plain radiography of the uninjured right side showed that Baumann’s angle and lateral capitellohumeral angle were 66 degrees and 45 degrees, respectively. The latest plain radiographs two years prior to the fourth injury showed a varus deformity of the left elbow, and Baumann’s angle and lateral capitellohumeral angle were 94 degrees and 58 degrees, respectively [9,10]. We considered that the lateral condylar fracture needed surgical treatment and that the multiple fractures of the same distal humerus were caused by the cubitus varus deformity. We explained our consideration to the parents and consent was obtained for surgical treatment of both the lateral condylar fracture and corrective osteotomy for the cubitus varus deformity to prevent future fractures.

**Figure 3:**
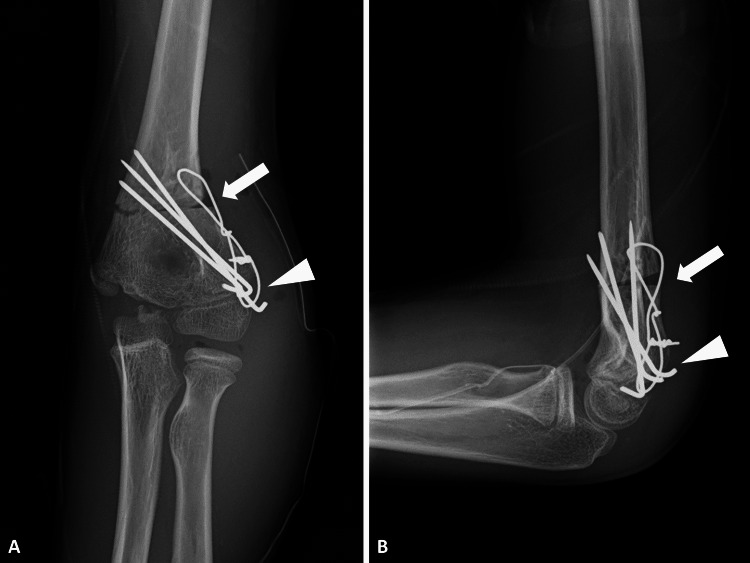
Plain radiography after operation of open reduction and corrective osteotomy. (A) Anteroposterior and (B) lateral views of the left elbow after operation. (Arrows: osteotomy site; Arrowheads: fracture site).

Surgical treatment was performed under supine position and general anesthesia on the same day of injury. Examination using a goniometer showed that the left elbow was 15 degrees varus compared to the right elbow. A direct lateral approach was used. The lateral ridge of the humerus was exposed and brachialis, extensors, and triceps were released under the periosteum. Proximal to the olecranon and coronoid fossa, two 1.5 mm wires were inserted from the lateral side as a guide of a 15 degrees lateral closing-wedge osteotomy using a sterile goniometer. Proximal to the osteotomy line, soft wire was inserted after making a hole using a 1.5 mm wire. Minimum incision of extensors from the fragment of the lateral condyle and anterior capsule was retracted. Hematoma at the fracture site was removed and open reduction was performed. Two 1.5 mm wires were inserted from the lateral condyle to the distal osteotomy site. Osteotomy using an oscillating saw was performed. After removing the wedged bone and contacting the distal and proximal end, the osteotomy site was fixed using the previous two wires. One wire was added and the proximal humerus, distal humerus, and lateral condyle were fixed using a tension band wiring technique. Postoperative plain radiography showed that Baumann’s angle and lateral capitellohumeral angle were 68 degrees and 60 degrees, respectively (Figures 3A, 3B).

**Figure 4:**
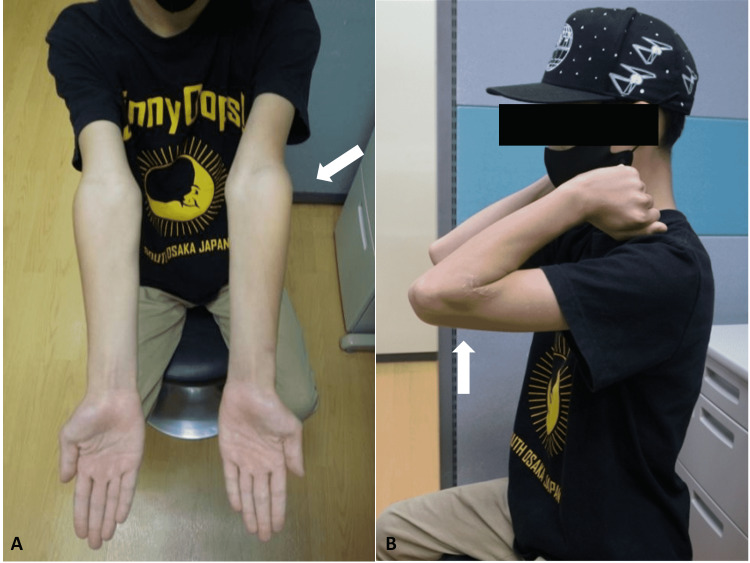
Photographs of the upper extremity at one and a half years postoperatively. (A) Gross appearance at elbow extension and (B) elbow flexion. (Arrows: injured side).

After the operation, the elbow was immobilized with a long arm splint for a week. A long arm cast was applied for five weeks. At six weeks postoperatively, after maturation of the callus at the osteotomy site, the cast was removed, and the range of elbow motion started. The patient’s postoperative course was uneventful. After nine months postoperatively, wires were removed under general anesthesia. At one and a half years postoperatively, the patient was asymptomatic and satisfied with the cosmetic issue of the elbow (Figures 4A, 4B).

**Figure 5:**
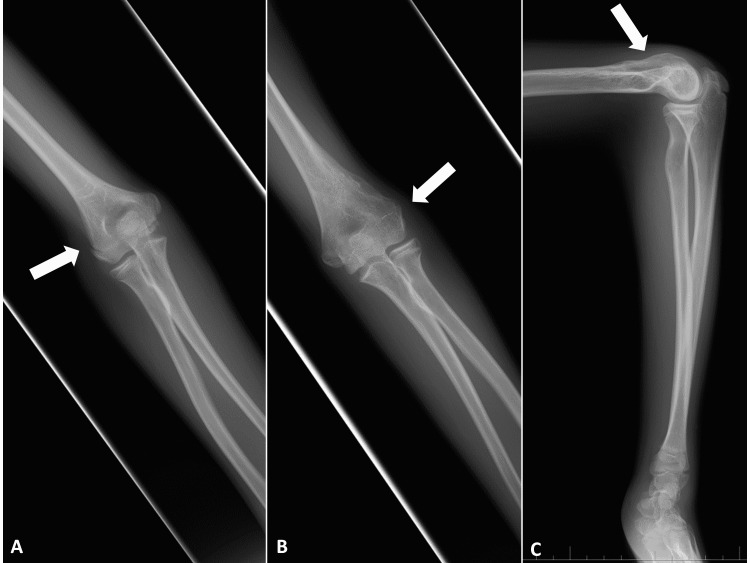
Plain radiography of the upper extremity at one and a half years postoperatively. (A) Anteroposterior view of the uninjured right elbow. (B) Anteroposterior and (C) lateral views of the left elbow. (Arrows: alignment of distal humerus).

Plain radiography showed that the coronal alignment was maintained. The closure of the physis at the distal humerus (which was earlier than that of the uninjured side) and no deformity, including fishtail deformity or osteonecrosis were noted (Figures 5A-5C).

There was no fracture during the postoperative period. The grip strengths of the right and left hands were 21.5 kg and 21.0 kg, respectively. The carrying angle was 5° in the right elbow and 5° in the left elbow. The ranges of motion of the right and left upper extremities were as follows: elbow flexion, 150° and 150°, respectively; elbow extension, 20° and 35°, respectively.

## Discussion

Traumatic cubitus varus deformity in the pediatric period can be caused by malunion after supracondylar humeral fracture, posttraumatic physeal stimulation with lateral overgrowth after lateral condylar fracture of the humerus, or early physeal closure after a physeal fracture of the distal humerus [1,4]. Pirone et al. reported that the occurrence of a cubitus varus deformity was 8.7% in patients with displaced extension-type supracondylar humeral fractures in childhood [3]. This deformity might cause later complications, tardy ulnar nerve palsy, posterolateral rotatory instability of the elbow, and second fractures of the distal humerus; the timing of the onset of these symptoms differs. Abe et al. reported that tardy ulnar nerve palsy occurred at an average of 15 years after fracture of the distal humerus [6]. O’Driscoll et al. reported that posterolateral rotatory instability occurred approximately two to three decades after the deformity happened [7]. For the second fracture, the average period after initial fracture was reported as 18 months (lateral condylar fracture; seven cases, fracture-separation of the entire distal humeral epiphysis; two cases by Takahara et al. [5]), 26 months (lateral condylar fracture; seven cases, supracondylar fracture; two cases by Park et al. [8]), and 32 months (lateral condylar fracture; six cases by Davids et al. [1]). In reports by Abe et al., four of 15 cases of patients with tardy nerve palsy suffered second fractures at an average of 24 months (5-60 months) after the initial fracture [6]. Thus, second fracture of the distal humerus occurs as the earliest of the three later complications.

Our patient suffered fractures of the same distal humerus four times in five years. The relationship between the occurrence of the second fracture and the extent of the cubitus varus deformity of the elbow is not clear. Davids et al. discussed pathomechanism of second lateral condylar fracture after cubitus varus deformity and described that posttraumatic cubitus varus alignment could increase both distraction and shear forces across the lateral condyle and cause subsequent lateral condylar fracture [1]. As the number of fracture at the same place was frequent, we selected an additional procedure, corrective osteotomy, to prevent sequential fractures and future complications and to decrease the total number of surgeries.

There are various methods concerning the corrective osteotomy for cubitus varus deformity. Takagi et al. reported a comparative study of supracondylar osteotomy of humerus to correct cubitus varus between three-dimensional osteotomy and simple coronal plane osteotomy [4]. They described that correction of internal rotation may not be necessary because it was difficult to maintain it in a three-dimensional osteotomy, and correction of malunion of extension in patients older than 10 years of age was necessary. In our case, there was a small extent of deformity in the sagittal plane. Therefore, we selected a simple lateral closing wedge osteotomy, because it would be possible to be performed in the same operative field through the lateral approach; therefore, it was feasible to perform both ORIF and osteotomy simultaneously.

Ippolito et al. reported a 23-year follow-up study of corrective osteotomy for posttraumatic cubitus varus in children [2]. Their results showed that two of 19 patients could maintain postoperative humeroulnar angle at the last follow-up. They concluded that the deformity followed either physeal injury or supracondylar fracture with damage to the physis secondary to the initial trauma, and the correction was not maintained. Fortunately, our case could keep the coronal alignment of the elbow at the time of physeal closure.

## Conclusions

Our case suffered fourth fracture at the same distal humerus due to posttraumatic cubitus varus deformity. We performed open reduction and internal fixation for lateral condyle fracture and lateral closing wedge osteotomy for cubitus varus deformity simultaneously. During follow-up, humeral alignment was maintained and there was no fracture. We think that two surgical procedures performed simultaneously can be indicated for the pediatric patients with the serial distal humeral fractures after cubitus varus deformity due to fracture.

## References

[1] Davids JR, Maguire MF, Mubarak SJ, Wenger DR (1994). Lateral condylar fracture of the humerus following posttraumatic cubitus varus. J Pediatr Orthop.

[2] Ippolito E, Moneta MR, D'Arrigo C (1990). Post-traumatic cubitus varus. Long-term follow-up of corrective supracondylar humeral osteotomy in children. J Bone Joint Surg Am.

[3] Pirone AM, Graham HK, Krajbich JI (1988). Management of displaced extension-type supracondylar fractures of the humerus in children. J Bone Joint Surg.

[4] Takagi T, Takayama S, Nakamura T, Horiuchi Y, Toyama Y, Ikegami H (2010). Supracondylar osteotomy of the humerus to correct cubitus varus: do both internal rotation and extension deformities need to be corrected?. J Bone Joint Surg Am.

[5] Takahara M, Sasaki I, Kimura T, Kato H, Minami A, Ogino T (1998). Second fracture of the distal humerus after varus malunion of a supracondylar fracture in children. J Bone Joint Surg Br.

[6] Abe M, Ishizu T, Shirai H, Okamoto M, Onomura T (1995). Tardy ulnar nerve palsy caused by cubitus varus deformity. J Hand Surg Am.

[7] O'Driscoll SW, Spinner RJ, McKee MD (2001). Tardy posterolateral rotatory instability of the elbow due to cubitus varus. J Bone Joint Surg Am.

[8] Park HW, Yang IH, Joo SY, Park KB, Kim HW (2007). Refractures of the upper extremity in children. Yonsei Med J.

[9] Shank CF, Wiater BP, Pace JL (2011). The lateral capitellohumeral angle in normal children: mean, variation, and reliability in comparison to Baumann's angle. J Pediatr Orthop.

[10] Worlock P (1986). Supracondylar fractures of the humerus. Assessment of cubitus varus by the Baumann angle. J Bone Joint Surg Br.

